# Supplementary material for: Modeling glioblastoma heterogeneity as a dynamic network of cell states
Source: Mol Syst Biol. 2021 Sep 16;17(9):e10105. doi: 10.15252/msb.202010105 (PMC8444284; doi:10.15252/msb.202010105)
Supplement: Supplementary file 5 — Source Data for Figure 3 [file MSB-17-e10105-s001.zip › Figure3A_sourcedata/GSEA_3065/nosigdb_state3.GseaPreranked.1622718426028/gsea_report_for_na_pos_1622718426028.html]

Report for na\_pos 1622718426028 [GSEA]

| GS  follow link to MSigDB | GS DETAILS | SIZE | ES | NES | NOM p-val | FDR q-val | FWER p-val | RANK AT MAX | LEADING EDGE || 1 | LARSSON\_STATE\_3 | Details ... | 86 | 1.00 | 3.88 | 0.000 | 0.000 | 0.000 | 100 | tags=100%, list=1%, signal=100% |
| 2 | LARSSON\_STATE\_2 | Details ... | 85 | 0.91 | 3.50 | 0.000 | 0.000 | 0.000 | 522 | tags=89%, list=6%, signal=94% |
| 3 | DOROSZKO\_U3180MG\_CLUSTER\_2 | Details ... | 463 | 0.70 | 3.22 | 0.000 | 0.000 | 0.000 | 1436 | tags=57%, list=16%, signal=65% |
| 4 | RICHARDS\_NOWAKOWSKI\_SCIENCE\_2017\_OPC\_UPREG | Details ... | 309 | 0.57 | 2.54 | 0.000 | 0.000 | 0.000 | 1240 | tags=42%, list=14%, signal=48% |
| 5 | IVY\_CT | Details ... | 140 | 0.59 | 2.43 | 0.000 | 0.000 | 0.000 | 1197 | tags=43%, list=14%, signal=49% |
| 6 | VANGRONINGEN\_MES | Details ... | 367 | 0.51 | 2.34 | 0.000 | 0.000 | 0.000 | 1321 | tags=38%, list=15%, signal=43% |
| 7 | RICHARDS\_ZHONG\_NATURE\_2018\_OPC\_UPREG | Details ... | 71 | 0.62 | 2.28 | 0.000 | 0.000 | 0.000 | 758 | tags=46%, list=9%, signal=50% |
| 8 | SEURAT\_CELLCYCLE | Details ... | 87 | 0.58 | 2.24 | 0.000 | 0.000 | 0.000 | 1440 | tags=40%, list=16%, signal=48% |
| 9 | RICHARDS\_NOWAKOWSKI\_SCIENCE\_2017\_ASTROCYTE\_UPREG | Details ... | 470 | 0.47 | 2.20 | 0.000 | 0.000 | 0.001 | 1222 | tags=36%, list=14%, signal=40% |
| 10 | ALLEN\_114\_OPCL1\_6MYT1 | Details ... | 16 | 0.83 | 2.18 | 0.000 | 0.000 | 0.001 | 684 | tags=63%, list=8%, signal=68% |
| 11 | RICHARDS\_NEFTEL\_CELL\_2019\_OPC | Details ... | 36 | 0.65 | 2.10 | 0.000 | 0.000 | 0.002 | 723 | tags=53%, list=8%, signal=57% |
| 12 | SUVA\_OPC\_PMID\_31327527 | Details ... | 36 | 0.65 | 2.07 | 0.000 | 0.000 | 0.003 | 723 | tags=53%, list=8%, signal=57% |
| 13 | GAROFANO\_IAVARONE\_NEU\_SUBTYPE | Details ... | 150 | 0.47 | 1.96 | 0.000 | 0.001 | 0.006 | 1978 | tags=39%, list=23%, signal=49% |
| 14 | TIISIG\_PMID\_32649888 | Details ... | 23 | 0.67 | 1.95 | 0.000 | 0.001 | 0.007 | 1113 | tags=43%, list=13%, signal=50% |
| 15 | GANDAL\_ALCOHOLADDICTION\_DN\_PMID29439242 | Details ... | 55 | 0.55 | 1.92 | 0.000 | 0.002 | 0.015 | 1180 | tags=38%, list=13%, signal=44% |
| 16 | RICHARDS\_VERHAAK\_CANCERCELL\_2010\_PRONEURAL | Details ... | 103 | 0.47 | 1.88 | 0.000 | 0.003 | 0.025 | 899 | tags=28%, list=10%, signal=31% |
| 17 | DOROSZKO\_U3013MG\_CLUSTER\_0 | Details ... | 26 | 0.63 | 1.87 | 0.000 | 0.003 | 0.025 | 344 | tags=42%, list=4%, signal=44% |
| 18 | IVY\_CTMVP | Details ... | 44 | 0.56 | 1.87 | 0.003 | 0.003 | 0.030 | 1890 | tags=57%, list=22%, signal=72% |
| 19 | NB\_KI\_CLUSTER8 | Details ... | 301 | 0.41 | 1.86 | 0.000 | 0.003 | 0.033 | 2091 | tags=40%, list=24%, signal=51% |
| 20 | RICHARDS\_NOWAKOWSKI\_SCIENCE\_2017\_VRG\_UPREG | Details ... | 375 | 0.39 | 1.78 | 0.000 | 0.007 | 0.078 | 1739 | tags=38%, list=20%, signal=46% |
| 21 | RICHARDS\_NOWAKOWSKI\_SCIENCE\_2017\_MGE\_IPC2\_UPREG |  | 472 | 0.38 | 1.77 | 0.000 | 0.007 | 0.081 | 1676 | tags=36%, list=19%, signal=42% |
| 22 | ALLEN\_111\_ASTROL1\_6FGFR3ETNPPL |  | 23 | 0.61 | 1.74 | 0.007 | 0.010 | 0.121 | 749 | tags=30%, list=9%, signal=33% |
| 23 | DOROSZKO\_U3054MG\_CLUSTER\_1 |  | 16 | 0.64 | 1.72 | 0.004 | 0.011 | 0.138 | 888 | tags=31%, list=10%, signal=35% |
| 24 | NB\_KI\_CLUSTER23 |  | 244 | 0.39 | 1.71 | 0.000 | 0.012 | 0.151 | 1308 | tags=30%, list=15%, signal=34% |
| 25 | DOROSZKO\_U3054MG\_CLUSTER\_5 |  | 93 | 0.44 | 1.71 | 0.000 | 0.011 | 0.151 | 1742 | tags=39%, list=20%, signal=48% |
| 26 | GANDAL\_SCHIZOPHRENIA\_DN\_PMID29439242 |  | 17 | 0.63 | 1.70 | 0.013 | 0.012 | 0.164 | 585 | tags=12%, list=7%, signal=13% |
| 27 | GAROFANO\_IAVARONE\_PPR\_SUBTYPE |  | 389 | 0.37 | 1.70 | 0.000 | 0.012 | 0.164 | 1606 | tags=31%, list=18%, signal=36% |
| 28 | RICHARDS\_ZHONG\_NATURE\_2018\_ASTROCYTES\_UPREG |  | 224 | 0.39 | 1.69 | 0.000 | 0.011 | 0.166 | 1162 | tags=33%, list=13%, signal=38% |
| 29 | DOROSZKO\_U3054MG\_CLUSTER\_0 |  | 54 | 0.48 | 1.67 | 0.003 | 0.014 | 0.206 | 1291 | tags=41%, list=15%, signal=47% |
| 30 | DOROSZKO\_PERIVASCULAR\_INVASION |  | 33 | 0.53 | 1.67 | 0.012 | 0.014 | 0.208 | 344 | tags=33%, list=4%, signal=35% |
| 31 | RICHARDS\_NOWAKOWSKI\_SCIENCE\_2017\_MGE\_RG1\_UPREG |  | 306 | 0.37 | 1.66 | 0.000 | 0.014 | 0.219 | 1167 | tags=28%, list=13%, signal=32% |
| 32 | RICHARDS\_NEFTEL\_CELL\_2019\_G2\_M |  | 43 | 0.49 | 1.66 | 0.000 | 0.014 | 0.225 | 1728 | tags=40%, list=20%, signal=49% |
| 33 | SINGH\_SOX2\_UPREGULATED\_TRANSCRIPTS\_PMID\_28122245 |  | 403 | 0.36 | 1.65 | 0.000 | 0.015 | 0.244 | 1978 | tags=33%, list=23%, signal=40% |
| 34 | DOROSZKO\_U3013MG\_CLUSTER\_2 |  | 201 | 0.38 | 1.65 | 0.000 | 0.015 | 0.259 | 1373 | tags=30%, list=16%, signal=35% |
| 35 | NB\_KI\_CLUSTER17 |  | 124 | 0.40 | 1.63 | 0.000 | 0.018 | 0.305 | 1138 | tags=39%, list=13%, signal=44% |
| 36 | DOROSZKO\_U3054MG\_CLUSTER\_2 |  | 230 | 0.37 | 1.62 | 0.000 | 0.018 | 0.318 | 1678 | tags=37%, list=19%, signal=45% |
| 37 | NB\_KI\_CLUSTER7 |  | 255 | 0.37 | 1.61 | 0.000 | 0.019 | 0.333 | 3260 | tags=47%, list=37%, signal=72% |
| 38 | NB\_KI\_CLUSTER14 |  | 206 | 0.37 | 1.61 | 0.000 | 0.019 | 0.339 | 1669 | tags=32%, list=19%, signal=39% |
| 39 | ANGIOGENESIS\_GO\_0001525 |  | 20 | 0.58 | 1.61 | 0.007 | 0.019 | 0.340 | 1448 | tags=55%, list=17%, signal=66% |
| 40 | SUVA\_G2M\_PMID\_31327527 |  | 43 | 0.49 | 1.61 | 0.008 | 0.018 | 0.342 | 1728 | tags=40%, list=20%, signal=49% |
| 41 | ALLEN\_116\_OLIGOL4\_6OPALIN |  | 21 | 0.57 | 1.60 | 0.025 | 0.020 | 0.369 | 1555 | tags=48%, list=18%, signal=58% |
| 42 | NB\_KI\_CLUSTER2 |  | 247 | 0.36 | 1.60 | 0.000 | 0.019 | 0.374 | 1711 | tags=34%, list=20%, signal=41% |
| 43 | LARSSON\_STATE\_5 |  | 98 | 0.41 | 1.58 | 0.000 | 0.021 | 0.410 | 357 | tags=42%, list=4%, signal=43% |
| 44 | RICHARDS\_VERHAAK\_CANCERCELL\_2010\_CLASSICAL |  | 109 | 0.39 | 1.57 | 0.000 | 0.025 | 0.463 | 1290 | tags=28%, list=15%, signal=33% |
| 45 | OLSEN\_SCPLIKE |  | 75 | 0.42 | 1.57 | 0.009 | 0.024 | 0.463 | 973 | tags=44%, list=11%, signal=49% |
| 46 | KRONA\_ROSEN\_WHITE\_MATTER\_INVASION |  | 175 | 0.36 | 1.56 | 0.000 | 0.025 | 0.475 | 1923 | tags=34%, list=22%, signal=42% |
| 47 | GANDAL\_BIPOLAR\_UP\_PMID29439242 |  | 57 | 0.43 | 1.53 | 0.010 | 0.030 | 0.547 | 1317 | tags=35%, list=15%, signal=41% |
| 48 | NB\_KI\_CLUSTER24 |  | 162 | 0.36 | 1.49 | 0.000 | 0.042 | 0.681 | 3349 | tags=48%, list=38%, signal=76% |
| 49 | WANG\_CLASSICAL\_PMID\_28697342 |  | 34 | 0.46 | 1.49 | 0.033 | 0.041 | 0.686 | 1033 | tags=24%, list=12%, signal=27% |
| 50 | RICHARDS\_ZHONG\_NATURE\_2018\_NPCS\_UPREG |  | 120 | 0.36 | 1.44 | 0.010 | 0.056 | 0.806 | 1548 | tags=36%, list=18%, signal=43% |
| 51 | ANASTASSIOU\_MULTICANCER\_INVASIVENESS\_PMID\_22208948 |  | 27 | 0.47 | 1.42 | 0.086 | 0.065 | 0.851 | 973 | tags=52%, list=11%, signal=58% |
| 52 | ALMSTEDT\_ALK |  | 48 | 0.42 | 1.42 | 0.037 | 0.065 | 0.858 | 1461 | tags=25%, list=17%, signal=30% |
| 53 | FELDMAN\_2020\_G1 |  | 146 | 0.33 | 1.39 | 0.003 | 0.079 | 0.913 | 1028 | tags=26%, list=12%, signal=29% |
| 54 | PARADA\_T1\_UP\_26461091 |  | 61 | 0.38 | 1.38 | 0.041 | 0.080 | 0.918 | 1624 | tags=38%, list=19%, signal=46% |
| 55 | ALLEN\_115\_OLIGOL4\_6MOBPCOL18A1 |  | 24 | 0.46 | 1.37 | 0.086 | 0.085 | 0.940 | 1555 | tags=42%, list=18%, signal=51% |
| 56 | KLING\_GBM\_PHENO\_PMID\_27667176 |  | 137 | 0.33 | 1.36 | 0.024 | 0.087 | 0.947 | 1289 | tags=28%, list=15%, signal=32% |
| 57 | DOROSZKO\_U3180MG\_CLUSTER\_1 |  | 16 | 0.51 | 1.36 | 0.118 | 0.088 | 0.950 | 1261 | tags=38%, list=14%, signal=44% |
| 58 | OUTERRADIALGLIA |  | 48 | 0.40 | 1.35 | 0.058 | 0.090 | 0.956 | 556 | tags=27%, list=6%, signal=29% |
| 59 | RICHARDS\_NOWAKOWSKI\_SCIENCE\_2017\_IN\_CTX\_CGE2\_UPREG |  | 223 | 0.31 | 1.35 | 0.010 | 0.091 | 0.958 | 1458 | tags=30%, list=17%, signal=36% |
| 60 | DOROSZKO\_U3054MG\_CLUSTER\_7 |  | 27 | 0.44 | 1.34 | 0.091 | 0.093 | 0.963 | 1299 | tags=26%, list=15%, signal=30% |
| 61 | RICHARDS\_NOWAKOWSKI\_SCIENCE\_2017\_NEN\_EARLY2\_UPREG |  | 379 | 0.29 | 1.34 | 0.000 | 0.093 | 0.963 | 2046 | tags=30%, list=23%, signal=38% |
| 62 | NB\_KI\_CLUSTER6 |  | 464 | 0.29 | 1.33 | 0.000 | 0.096 | 0.970 | 2629 | tags=38%, list=30%, signal=52% |
| 63 | FELDMAN\_2020\_LATEG1 |  | 89 | 0.35 | 1.33 | 0.053 | 0.099 | 0.974 | 915 | tags=25%, list=10%, signal=27% |
| 64 | POLLEN\_OUTERRADIALGLIA\_PMID\_26406371 |  | 48 | 0.40 | 1.32 | 0.052 | 0.102 | 0.978 | 556 | tags=27%, list=6%, signal=29% |
| 65 | SUVA\_AC\_PMID\_31327527 |  | 32 | 0.42 | 1.32 | 0.082 | 0.101 | 0.978 | 1118 | tags=47%, list=13%, signal=54% |
| 66 | DALMO\_BARCODENUMBER\_UNPUB |  | 187 | 0.30 | 1.29 | 0.018 | 0.118 | 0.989 | 994 | tags=26%, list=11%, signal=28% |
| 67 | RICHARDS\_NEFTEL\_CELL\_2019\_AC |  | 32 | 0.42 | 1.29 | 0.145 | 0.119 | 0.991 | 1118 | tags=47%, list=13%, signal=54% |
| 68 | RICHARDS\_ZHONG\_NATURE\_2018\_MICROGLIA\_UPREG |  | 191 | 0.29 | 1.27 | 0.034 | 0.137 | 0.995 | 1842 | tags=37%, list=21%, signal=45% |
| 69 | RICHARDS\_NOWAKOWSKI\_SCIENCE\_2017\_IPC\_NEN1\_UPREG |  | 218 | 0.29 | 1.26 | 0.022 | 0.138 | 0.995 | 1916 | tags=31%, list=22%, signal=39% |
| 70 | NB\_KI\_CLUSTER18 |  | 69 | 0.35 | 1.26 | 0.101 | 0.144 | 0.995 | 973 | tags=32%, list=11%, signal=36% |
| 71 | DEPRETER\_LOW\_NB\_RISK |  | 18 | 0.45 | 1.25 | 0.198 | 0.150 | 0.995 | 778 | tags=28%, list=9%, signal=30% |
| 72 | GANDAL\_BIPOLAR\_DN\_PMID29439242 |  | 18 | 0.45 | 1.25 | 0.175 | 0.149 | 0.995 | 778 | tags=22%, list=9%, signal=24% |
| 73 | GANDAL\_IBD\_UP\_PMID29439242 |  | 54 | 0.36 | 1.24 | 0.157 | 0.151 | 0.995 | 1678 | tags=35%, list=19%, signal=43% |
| 74 | TISIG\_PMID\_32649888 |  | 32 | 0.40 | 1.24 | 0.163 | 0.151 | 0.995 | 1461 | tags=38%, list=17%, signal=45% |
| 75 | ALLEN\_112\_ASTROL1FGFR3MT1G |  | 25 | 0.43 | 1.24 | 0.180 | 0.152 | 0.997 | 772 | tags=36%, list=9%, signal=39% |
| 76 | RICHARDS\_NOWAKOWSKI\_SCIENCE\_2017\_NIN3\_UPREG |  | 137 | 0.30 | 1.21 | 0.106 | 0.180 | 0.998 | 1047 | tags=22%, list=12%, signal=24% |
| 77 | SUVA\_NPC1\_PMID\_31327527 |  | 34 | 0.37 | 1.19 | 0.210 | 0.193 | 0.999 | 999 | tags=29%, list=11%, signal=33% |
| 78 | NB\_KI\_CLUSTER20 |  | 16 | 0.44 | 1.19 | 0.247 | 0.192 | 0.999 | 3114 | tags=63%, list=36%, signal=97% |
| 79 | SEGERMAN\_MULTITHERAPY\_RESISTANCE\_FIG5A\_PMID\_27974212 |  | 22 | 0.41 | 1.19 | 0.219 | 0.193 | 0.999 | 2301 | tags=41%, list=26%, signal=55% |
| 80 | RICHARDS\_NEFTEL\_CELL\_2019\_NPC1 |  | 34 | 0.37 | 1.18 | 0.203 | 0.194 | 0.999 | 999 | tags=29%, list=11%, signal=33% |
| 81 | RICHARDS\_NOWAKOWSKI\_SCIENCE\_2017\_NIN5\_UPREG |  | 254 | 0.26 | 1.17 | 0.066 | 0.207 | 0.999 | 1607 | tags=28%, list=18%, signal=33% |
| 82 | GANDAL\_SCHIZOPHRENIA\_UP\_PMID29439242 |  | 72 | 0.31 | 1.17 | 0.160 | 0.207 | 0.999 | 1358 | tags=28%, list=16%, signal=33% |
| 83 | NB\_KI\_CLUSTER15 |  | 147 | 0.28 | 1.16 | 0.135 | 0.211 | 0.999 | 3221 | tags=42%, list=37%, signal=66% |
| 84 | NB\_KI\_CLUSTER5 |  | 86 | 0.30 | 1.16 | 0.183 | 0.216 | 1.000 | 949 | tags=26%, list=11%, signal=28% |
| 85 | PARADA\_T2\_UP\_26461091 |  | 62 | 0.32 | 1.15 | 0.204 | 0.231 | 1.000 | 1624 | tags=34%, list=19%, signal=41% |
| 86 | RICHARDS\_NOWAKOWSKI\_SCIENCE\_2017\_IN\_STR\_UPREG |  | 173 | 0.27 | 1.14 | 0.135 | 0.241 | 1.000 | 1804 | tags=29%, list=21%, signal=36% |
| 87 | ALLEN\_113\_ASTROL1FGFR3FOS |  | 24 | 0.37 | 1.09 | 0.330 | 0.319 | 1.000 | 855 | tags=38%, list=10%, signal=41% |
| 88 | RICHARDS\_LIDDELOW\_NATURE\_2017\_A1\_REACTIVE\_ASTROCYTES |  | 19 | 0.40 | 1.08 | 0.338 | 0.319 | 1.000 | 2018 | tags=53%, list=23%, signal=68% |
| 89 | RICHARDS\_NOWAKOWSKI\_SCIENCE\_2017\_NIN1\_UPREG |  | 143 | 0.26 | 1.08 | 0.246 | 0.317 | 1.000 | 1269 | tags=24%, list=14%, signal=27% |
| 90 | DOROSZKO\_U3054MG\_CLUSTER\_6 |  | 110 | 0.27 | 1.06 | 0.296 | 0.352 | 1.000 | 1728 | tags=29%, list=20%, signal=36% |
| 91 | RICHARDS\_VERHAAK\_CANCERCELL\_2010\_MESENCHYMAL |  | 97 | 0.27 | 1.02 | 0.378 | 0.429 | 1.000 | 731 | tags=16%, list=8%, signal=18% |
| 92 | DOROSZKO\_U3013MG\_CLUSTER\_1 |  | 36 | 0.32 | 1.02 | 0.425 | 0.443 | 1.000 | 305 | tags=11%, list=3%, signal=11% |
| 93 | DOROSZKO\_U3054MG\_CLUSTER\_3 |  | 191 | 0.23 | 0.98 | 0.487 | 0.528 | 1.000 | 1865 | tags=25%, list=21%, signal=31% |
| 94 | KRONA\_ROSEN\_PERIVASCULAR\_INVASION |  | 44 | 0.29 | 0.96 | 0.499 | 0.572 | 1.000 | 2112 | tags=34%, list=24%, signal=45% |
| 95 | GANDAL\_IBD\_DN\_PMID29439242 |  | 20 | 0.33 | 0.92 | 0.568 | 0.674 | 1.000 | 1738 | tags=45%, list=20%, signal=56% |
| 96 | NB\_KI\_CLUSTER9 |  | 18 | 0.32 | 0.88 | 0.628 | 0.759 | 1.000 | 1472 | tags=22%, list=17%, signal=27% |
| 97 | NB\_KI\_CLUSTER22 |  | 126 | 0.20 | 0.81 | 0.929 | 0.880 | 1.000 | 1811 | tags=24%, list=21%, signal=30% |
| 98 | PARADA\_T1\_DN\_26461091 |  | 36 | 0.24 | 0.78 | 0.836 | 0.904 | 1.000 | 2592 | tags=42%, list=30%, signal=59% |
| 99 | PARADA\_T2\_DN\_26461091 |  | 38 | 0.18 | 0.61 | 0.979 | 0.987 | 1.000 | 2117 | tags=29%, list=24%, signal=38% |
Table: Gene sets enriched in phenotype **na **[plain text format]****
